# Improving Fishing Pattern Detection from Satellite AIS Using Data Mining and Machine Learning

**DOI:** 10.1371/journal.pone.0158248

**Published:** 2016-07-01

**Authors:** Erico N. de Souza, Kristina Boerder, Stan Matwin, Boris Worm

**Affiliations:** 1 Big Data Analytics Institute, Faculty of Computer Science, Dalhousie University, Halifax, NS, Canada; 2 Biology Department, Dalhousie University, Halifax, NS, Canada; 3 Institute of Computer Science, Polish Academy of Sciences, Warsaw, Poland; Aristotle University of Thessaloniki, GREECE

## Abstract

A key challenge in contemporary ecology and conservation is the accurate tracking of the spatial distribution of various human impacts, such as fishing. While coastal fisheries in national waters are closely monitored in some countries, existing maps of fishing effort elsewhere are fraught with uncertainty, especially in remote areas and the High Seas. Better understanding of the behavior of the global fishing fleets is required in order to prioritize and enforce fisheries management and conservation measures worldwide. Satellite-based Automatic Information Systems (S-AIS) are now commonly installed on most ocean-going vessels and have been proposed as a novel tool to explore the movements of fishing fleets in near real time. Here we present approaches to identify fishing activity from S-AIS data for three dominant fishing gear types: trawl, longline and purse seine. Using a large dataset containing worldwide fishing vessel tracks from 2011–2015, we developed three methods to detect and map fishing activities: for trawlers we produced a Hidden Markov Model (HMM) using vessel speed as observation variable. For longliners we have designed a Data Mining (DM) approach using an algorithm inspired from studies on animal movement. For purse seiners a multi-layered filtering strategy based on vessel speed and operation time was implemented. Validation against expert-labeled datasets showed average detection accuracies of 83% for trawler and longliner, and 97% for purse seiner. Our study represents the first comprehensive approach to detect and identify potential fishing behavior for three major gear types operating on a global scale. We hope that this work will enable new efforts to assess the spatial and temporal distribution of global fishing effort and make global fisheries activities transparent to ocean scientists, managers and the public.

## 1 Introduction

A common challenge in ecology is the mapping of dynamic patterns of human activity across vast areas in order to understand and track their ecosystem impacts on regional and global scales [1–3]. While important from a scientific perspective, there are also many other obvious applications, including the monitoring of marine fisheries and the enforcement of spatial management measures, such as marine protected areas (MPAs), ecologically and biologically sensitive areas (EBSAs) as well as fisheries closure zones. While the reception range of coastal monitoring tools such as tower-based applications (tAIS, radar) is limited to inshore areas, long-range tools such as satellite-based AIS (Automatic Identification System) and VMS (Vessel Monitoring System) provide insight into vessel movements elsewhere. VMS was specifically designed to monitor commercial fisheries while AIS was intended as a safety feature to avoid vessel collisions under low visibility. While the use of VMS devices is mandated only for some fleets in individual nations, the International Maritime Organization (IMO) has made the carrying of an AIS transponder mandatory for all vessels larger than 300 gross tons or carrying passengers (SOLAS Chapter V). In addition, national regulations may include other vessel types, such as per recent requirements by the European Union that all fishing vessels bigger than 15m must carry an AIS device [4]. Both VMS and AIS feature on-board transmitters linked to the vessel’s GPS to receive and transmit exact position in time and space on long-range radio frequencies to either coastal ground stations or satellites. In the case of AIS, data are also transmitted to other ships in the area that carry the device. VMS usually transmits in time intervals varying from one to several hours, satellite-based AIS (S-AIS) transmissions can be as frequent as every few seconds, enabling the monitoring of fine-scale vessel behavior and movement patterns.

Several attempts have been made to use VMS and AIS data to understand fishing vessel behavior, for example by using simple presence/absence or vessel speed [5] [6]. While speed can be a useful indicator of vessel activity, operational speeds while fishing vary greatly for different fishing gear types such as trawls, longlines or nets. More sophisticated algorithms differentiating fishing from non-fishing activity for different fleets and gears are needed to properly capture and represent the characteristics of the various fishing methods, as stated previously by Natale *et al*. [4] We develop and present such algorithms here, then assess their accuracy in correctly identifying individual fishing events or ‘sets’ by comparing against expert-labeled data. Finally, we briefly chart potential applications in marine ecology, conservation, and fisheries management.

## 2 Methods

### 2.1 Data Sets

**Fig 1:**
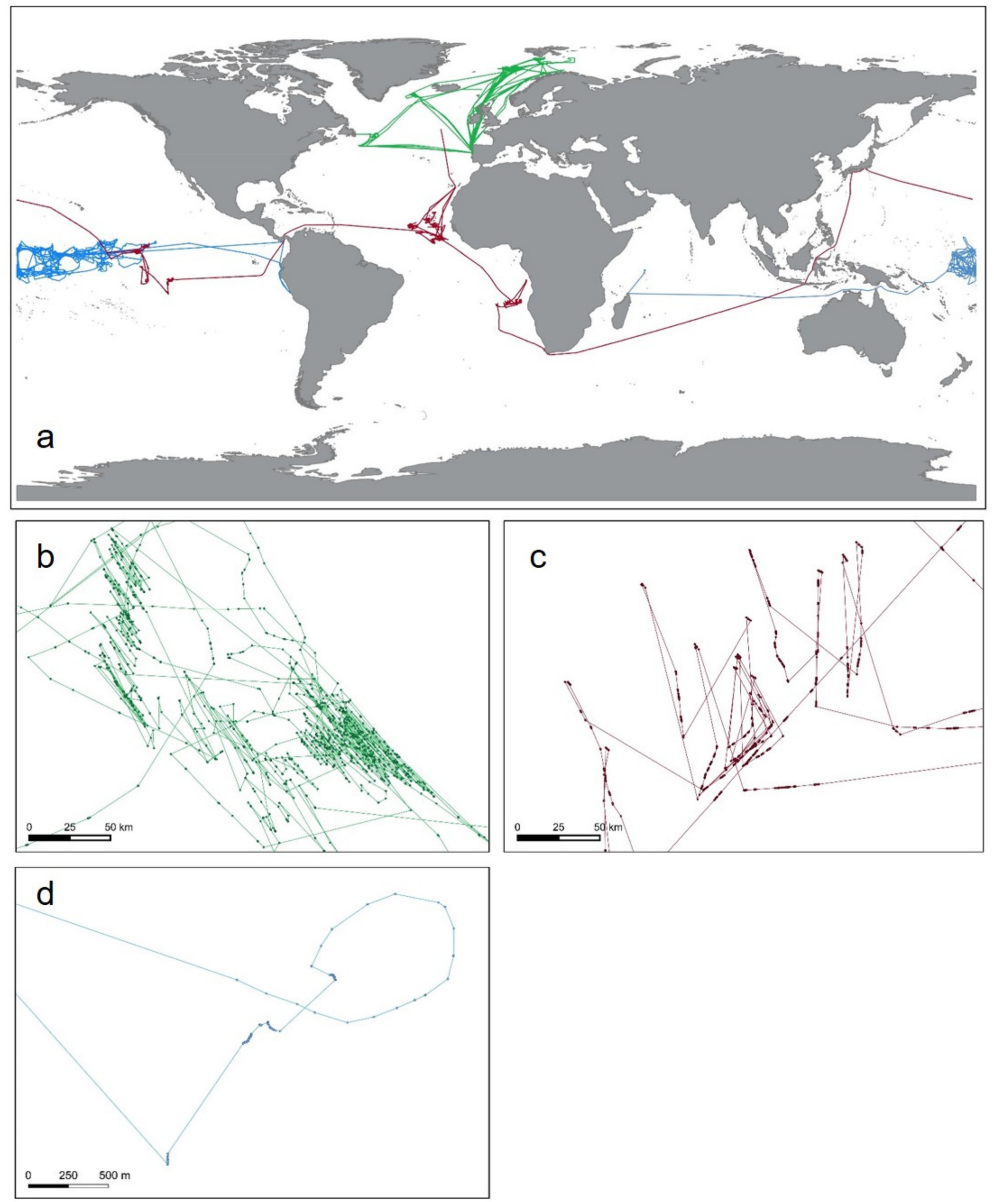
Presentation of raw S-AIS tracks for three individual vessels using different fishing gear types in global overview (A) and more fine-scale representations of potential fishing behavior for a trawler (green, B), longliner (red, C) and purse seiner (blue, D). Dots represent individual S-AIS signal detections, lines interpolated tracks. Note the global-range behavior of longliners, and the more regional basin-wide operations of purse seine and trawl vessels. Map data by Natural Earth.

This work is based on a database containing global S-AIS data obtained from AIS-enabled communication satellites since January 2011 until October 2015. Data were obtained under research licence from exactEarth (http://www.exactearth.com/products/exactais). A representation of three several-year tracks and examples of fishing activity patterns for trawling, longlining and purse seining is given in Fig 1. Individual tracks for known trawl, longliner and purse seine fishing vessels were extracted from different regions representing vessels from different nations operating in various parts of the oceans at scales from coastal fishing grounds to circumnavigating the globe.

The trawler data contained an initial sample of 83 vessels operating in the North Pacific and corresponds to 217,860 data points collected in July 2013 used for algorithm development and training. For comparison and testing on a global scale, a second trawler data set, composed of seven vessels operating from January 2011 until October 2015 across various ocean basins was selected. These tracks were much longer than those in the North Pacific, totaling 884,478 data points.

Analyses for longliners comprised data from 16 vessels operating across all major ocean basins from June 2012 until December 2013, corresponding to 573,204 data points.

Data on 7 purse seine vessels comprised 399,545 points from January 2011 until October 2015 representing long-range operations in various areas of the world.

### 2.2 Definition of Fishing Activity by Gear Type

#### 2.2.1 Trawler

Trawling involves dragging one or more nets behind a fishing vessel either on the sea floor (bottom trawling) or in the water column (pelagic or midwater trawling). While trawling, fishing vessels usually slow down and aim to maintain a constant speed to keep the strain on the dragged net as even as possible. Duration of trawling operations depends mostly on the density of the prey and can last from a few minutes up to several hours. The typical length of a trawl will vary between 3 and 5 hours [7]. Here, trawling activity is defined from the moment the net is deployed to when it is retrieved. Trawls are often characterized by slow, steady speeds between 2.5 and 5.5 knots. These speed thresholds were determined directly from the distribution of the AIS speed data and correspond to similar values obtained from literature [8–11].

#### 2.2.2 Longliner

Longlining involves the setting of fishing lines (up to 100 km length) equipped with several hundred to several thousands of hooks [12]. Lines can be deployed at various depths with the use of floats and horizontal lines extending to deeper waters. To set the line, the vessel travels only slightly slower than its steaming speed while the line is set. After the last hook is in the water, the line is left in the water for some hours (‘soak time’). During this time, the vessel either drifts slowly with the line or sets other lines in the vicinity. To haul the line the vessel reverses and steams back along the line. The whole operation can take up to a day. Speed while hauling is kept mostly constant but can vary according to catch and number of crew working. The time to set a longline depends on the length of the line and the number of crew working it, but the median set time estimated from the 16 vessels used in our analyses was 6.5 hours. Here a longline set is considered to start with setting of the longline and to end with retrieval of the last hooks. Characteristics used for identification of longline sets comprise spatial-temporal movement patterns in a very restricted area.

#### 2.2.3 Purse seiner

Purse seines are long nets deployed hanging vertically from floats around schooling fish on or near the surface by the vessel or by a separate skiff. To avoid fish escaping the setting of the seine needs to happen quickly and is done at high speeds averaging around 10 knots. Once the net encircles the school completely, the bottom of the net is pulled shut and the net hauled. Drifting with the net attached, the fish are then retrieved and transferred to the vessel. The duration of this process depends on the amount of catch and can vary from one to several hours [13].

For the purpose of this work, a purse seine set is defined as the time the net is closed around the fish to the end of the fish bailing operation when the net is lifted out of the water. During this time the purse seine vessel stays more or less stationary and speed over ground is generally slow, ranging around 2.5 knots and less. This threshold was determined based on speed distributions of the AIS data as well as observations from literature [14, 15].

### 2.3 Data Labeling and Pre-processing

All vessel tracks were classified and pre-labeled as potential fishing and non-fishing events by an expert based on information on fisheries characteristics as obtained from literature, analyses of the tracks (speed and movement profiles by gear type, flag, vessel size and area of operation), personal interviews with fishermen and fisheries on-board observers and comparisons to speed and movement profiles from observer data for the Northeast Atlantic. Characteristics include speed over ground, change of direction within a defined area, spatial-temporal movement patterns, operational time and duration of the fishing event. The testing of the algorithms against expert-labeled data was chosen because suitable observer or logbook data for the fleets and time period examined were unavailable to us Expert judgement on vessel behavior based on the aforementioned characteristics might be a conservative approach, as some fishing events will be missed.

In order to improve fishing activity prediction, for each data point we calculated whether it occurred during night or day. In order to estimate the amount of sun light available in a region of the world during a certain UTC-based time, the R package solaR [16] was used, with positive values for sunlight marking the day, zeroes marking the night. To avoid irregular vessel movement patterns very close to shore and in port a 10 km boundary around shorelines was established. The calculation requires the computation of the Haversine distance between each vessel track point and all the points in the shoreline data provided by Natural Earth [17] to establish the minimum distance to shore. This process is computationally expensive and to improve the quality of the calculation, it was decided to use the parallel capabilities provided by the code available in [18].

### 2.4 Algorithm Testing

The algorithms presented here were tested against expert-labeled fishing vessel tracks, separating fishing and non-fishing activity based on observations from operational data, expert knowledge and comparison to other tracking data. Accuracies presented are based on these comparisons.

Each algorithm proposed has different assumptions: the HMM assumes that the user will have part of the data available for training, while the Data Mining (DM) and filtering approaches do not require training. These differences determine how these approaches are tested: the HMM algorithm (applied for trawlers) uses Monte Carlo experiments to measure how it behaves with time variation, and the DM approach uses all data available for testing against expert-labeled data (used for longliner and purse seiner). For the trawling activity the HMM algorithm was tested with a Monte Carlo simulation using the implementation provided by the R package DMwR [19]. North Pacific trawler data set was used for this purpose. This data was the first data set that was available for development and testing of the HMM approach and offered a high variation of vessel behaviors within shorter tracks covering one month of data (July 2013). To ensure the applicability of the HMM trained on this data set, the HMM was subsequently applied and tested on a second data set containing multi-year trawler tracks operating in various parts of the ocean on regional and global scale. The Monte Carlo Simulation partitioned the data in 20 segments; each of these segments was trained with anywhere from 25,000 data to 130,000 points. All tests predicted 100,000 points in the future. The Monte Carlo simulations did not consider the 10 km threshold, as the HMM uses only speed as input. We decided to execute tests with fixed-size windows for training and test to avoid possible overfitting. DMwR [19] package randomly selects 20 data windows according to the user specification for training and test data, where the entire window is dislocated once the training and testing is done, and its respective statistical results are stored. The results of the testing are represented as Prediction (F for fishing) and Prediction (NF for non-fishing) in Tables 1 and 2 and were calculated based on [20]. These two metrics give an estimate of how well the algorithm predicts the desired class using unseen data.

**Table 1:** Performance measures for the worldwide trawl dataset. NF stands for probable non-fishing and F for probable fishing events. Sensitivity is related with non-fishing detection, and specificity with fishing detection. The column Stat. Diff. Fish Effort shows the t-test statistical comparison (*p*-value) between the predicted fishing effort time calculated from the algorithm’s labels and the expert’s labels. The asterisk indicates a significant difference.

Track ID	Track Size	Accuracy	Prediction (F)	Prediction (NF)	Sensitivity	Specificity	% of Fish Activity	Stat. Diff. Fish Effort
1	38258	0.75	0.47	0.96	0.90	0.71	0.42	0.84
2	254323	0.84	0.83	0.85	0.93	0.69	0.70	0.21
3	93670	0.83	0.82	0.95	0.99	0.40	0.89	0.11
4	56287	0.87	0.89	0.80	0.94	0.69	0.77	0.04*
5	55034	0.92	0.51	0.98	0.82	0.93	0.14	0.21
6	285407	0.57	0.28	0.93	0.84	0.51	0.55	0.09
7	101499	0.76	0.01	1.00	1.00	0.76	0.24	0.32
**Median±SD**		0.83±0.11	0.51±0.32	0.95±0.07	0.93±0.07	0.68±0.17		

**Table 2:** Performance measures for the 16 longliner vessels in different oceans. NF stands for probable non-fishing and F for probable fishing events. Sensitivity is related with non-fishing detection, and specificity with fishing detection. The column Stat. Diff. Fish Effort shows the t-test statistical comparison (*p*-value) between the predicted fishing effort time calculated from the algorithm’s labels and the expert’s labels. Two of the vessels could not be measured because they did not have any labeled fishing activity. The asterisk indicates a significant difference.

Track ID	Track Size	Accuracy	Prediction (NF)	Prediction (F)	Sensitivity	Specificity	% of Fish Activity	Stat. Diff. Fish Effort
1	7935	0.46	0.95	0.25	0.35	0.93	0.70	0.29
2	25558	0.80	0.56	0.87	0.52	0.88	0.80	0.08
3	9642	0.65	0.85	0.57	0.45	0.91	0.71	0.62
4	35258	0.89	0.70	0.93	0.71	0.93	0.81	0.67
5	34993	0.87	0.82	0.89	0.66	0.95	0.79	0.28
6	42566	0.89	0.83	0.90	0.54	0.98	0.88	0.35
7	25287	0.76	0.81	0.73	0.59	0.89	0.68	0.67
8	96314	0.54	0.97	0.48	0.22	0.99	0.87	0.05 *
9	123686	0.89	0.89	0.90	0.81	0.94	0.67	0.54
10	128668	0.74	0.96	0.66	0.49	0.98	0.75	0.82
11	2070	0.54	0.81	0.50	0.20	0.94	0.86	0.80
12	1452	0.95	0.95		1.00	0.00	0.00	NA
13	12405	0.71	0.93	0.43	0.68	0.82	0.44	0.34
14	18169	0.86	0.80	0.87	0.55	0.96	0.83	0.67
15	6421	0.99	0.99		1.00	0.00	0.00	NA
16	2780	0.88	0.93	0.80	0.89	0.86	0.36	0.30
**Median±SD**		0.83±0.15	0.87±0.11	0.77±0.21	0.57±0.24	0.93±0.04		

## 3 Data Analysis

### 3.1 HMM and Observation Variable Choice for Trawlers

Hidden Markov Models (HMM) represent a probability distribution over a sequence of points [21]. It is assumed that an observation at time *t* was generated by a hidden state *S*_*t*_. The second assumption is that given the value in *S*_*t*−1_, the value of *S*_*t*_ is independent of all previous states to *t* − 1. Assuming that the observed variable is defined by *Y*_*t*_ in time *t*, and states *S*_*t*_ are defined as classes {‘F’,‘N’}, then the Markov Model is factored in the following way:

$$\mathrm{Pr}\left(S_{1:T}|Y_{1:T}\right)=\mathrm{Pr}\left(S_{1}\right)\mathrm{Pr}\left(Y_{1}|S_{1}\right)\prod_{t=2}^{T}\mathrm{Pr}\left(Y_{t}|S_{t}\right)\mathrm{Pr}\left(S_{t}|S_{t-1}\right)$$ (1)

where *Pr*(*S*_*t*_|*S*_*t*−1_) represents the the transition matrix giving the probability of a state being changed to another state. In the case of fishing activity, it will represent the probability of changing the vessels’ state from fishing to non-fishing, and vice-versa. This transition matrix can be estimated directly from the distribution of fishing and non-fishing labels defined in the data set. Pr(*Y*_*t*_|*S*_*t*_) represents the probability of an observed variable occurring associated with a state *S*_*t*_ in time *t*. *T* represents the last speed read in the data set.

In order to build a successful HMM model it is necessary to define which parameters offer the best chance to identify the correct hidden states (fishing or non-fishing). Since speed is a key feature in all ecological work associated with HMM’s of animal movement [22], our work also implements an HMM based on speed.

**Fig 2:**
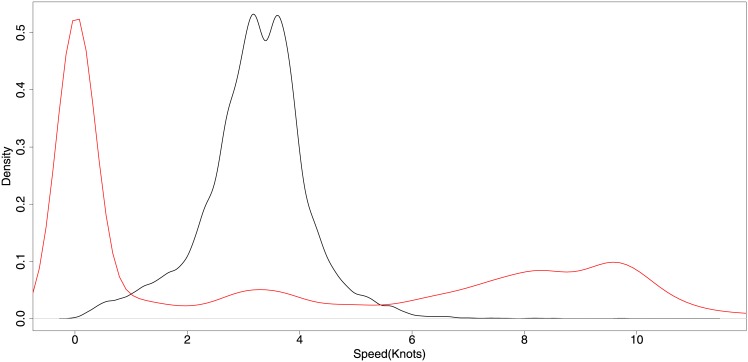
Speed distributions for trawlers during fishing and non-fishing activities. The red line represents probable non-fishing activity, and the black line probable fishing activity.

Fig 2 shows the speed distribution for vessels engaged in potential fishing or non-fishing activities (such as steaming, searching and anchoring). These results are comparable to those presented by others for mobile towed gear [23].

### 3.2 Data Mining Approach for Fishing Detection with Longliner

**Fig 3:**
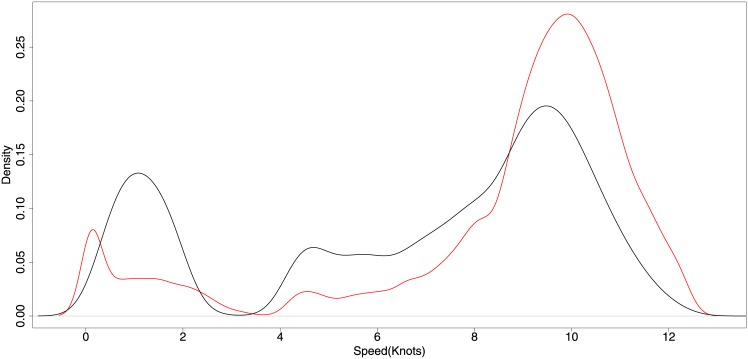
Speed distributions for longliners during fishing and non-fishing activities. The red line represents probable non-fishing activity, and the black line probable fishing activity.

The same HMM approach cannot be directly applied to the longliner data set as the speed distribution does not follow a clear pattern as seen in the trawler data (Fig 3). Several parameters contained in the data were tested as potential classifiers, but none proved sufficient to describe fishing and non-fishing activity patterns for longlining.

Therefore we opted to develop an alternative approach similar to what biologists have used in studying animal movement tracks. It has been shown that human fishermen tend to show similar movement patterns as animal predators simply because this is the most efficient method to search for and locate prey [15]. Building on this, we decided to use a segmentation technique traditionally applied to animal predators. The Lavielle’s segmentation algorithm [24, 25] is widely used by biologists to segment animal tracks in order to identify possible variations in their habitat use.

The Lavielle’s algorithm finds the best segmentation of a time series assuming that it is built by *K* segments defined by the user. The algorithm is not originally designed to work with GPS coordinates, but instead it will segment *any* regular time series data. Before segmentation the whole track was regularized in time to remove gaps in the GPS readings. The regularization assumes equal separation of seven hours between each GPS reading. The separation based on seven hours was defined based on the average time that longliners spent fishing as estimated from the labeled data set.

Lavielle’s algorithm searches for a minimum contrast estimator in a problem of change-points estimation, which suggests that Lavielle’s algorithm is a drift detector in a time series. The implementation used required the definition of *K*, which is the number of segments desired by the user as algorithm input, and it was estimated as 70 segments based on various tests. More information about the implementation used may be found in adehabitat package [26] in R.

For each of the segments a second algorithm was used to detect if the segment is composed of straight or curved lines. For each segment, the algorithm calculates the cosine of the relative angle between two consecutive points and calculates an average for all points in the segment. If the average cosine returns a value above 0.8 or below -0.8, it indicates that the whole segment is formed by a straight-line movement. All segments that contain the majority of their points classified as straight lines receive a label of non-fishing activity. The segments presenting curved movements are separated to execute a third algorithm to further filter non-fishing activity.

Once the curved segments are separated it is possible to analyze each point to remove possible non-fishing activity classified as fishing. Since the entire curved segment is considered fishing from the straight line algorithm detection, many non-fishing activities will be automatically assumed as fishing. In order to reduce this type of error, two other algorithms are combined to extract these false alarms: First-Passage Time algorithm (FPT) [27] and Utilization Distribution algorithm (UD) [28].

The FPT algorithm [27, 29] uses Brownian Motion theory to find areas where the patterns appear in a trajectory. According to [26], “for a given scale *r* it is defined as the time required by animals to pass through a circle of radius *r*”. This means that the FPT algorithm searches for the minimum radius *r* that contains multiple passes of the animal/vessel in the same region. One problem with the approach is that, depending on how long the track is, it does not restrict the size of *r*, which could result in a longer search for the correct radius. Fortunately, the movement of the vessels inside the curved segments is very restricted, which makes the search for *r* feasible. After some tests, we found that *r* using 30 different radii uniformly distributed varying from 0.1 to 1, offer a acceptable accuracy for FPT. Fauchald and Tveraa [27] extended the FPT algorithm to compute the variance of the *log*(*FPT*), which should be high for scales where vessels have multiple passes. As a threshold for our algorithm, if the *Var*(*log*(*FPT*)) ≤ 0.1 it is a straight line and these points are labeled as non-fishing.

To reduce possible false alarms, the Utilization Distribution algorithm (UD) [28] was used in addition. The UD is defined as a probability distribution [30] using only the longitude and latitude features. In order to estimate this distribution, a kernel method clustering algorithm is used in the coordinate parameters. The idea is to use a bivariate *kernel function* as a distance metric in each GPS location to find the cluster centers. The adehabitat implementation uses by default the normal kernel function, and we did not change this parameter. This work uses the UD estimations to correct wrong predictions of points wrongly classified as fishing activity to non-fishing activity.

The combination of FPT and UD within the curved data segments offers an extra 1% to 2% accuracy improvement and a reduction of non-fishing activity false alarm comparing to the expert labels.

### 3.3 Filtering Approach for Purse Seiner Fishing Detection

**Fig 4:**
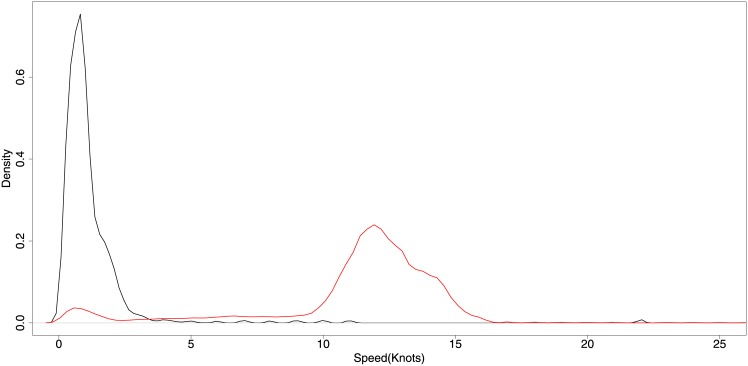
Speed distribution for Purse Seiners. The red line indicates the probable non-fishing activity labeled by the expert, while the black line is the probable fishing activity. This distribution considers only the speeds reported by the vessels 10 km from shore, and during day time.

Fishing activity detection for Purse Seiners builds on two assumptions based on literature data (see [14]), personal communication with fisheries observers working on board of various purse seiners and observations from the AIS data. Firstly, the majority of purse seiners do not fish at night, with rare exceptions that are not considered here. Second, that the fishing pattern consists of two main activity patterns, namely the setting of the net at high speeds and the drifting while hauling in the net and retrieving the fish at very low speeds. While the setting of the net is a very short activity that may not be represented in the data due to insufficient satellite coverage, the hauling and bailing can take up to several hours and is thus used to detect and classify potential fishing activity. Using the abovementioned day/night classifier, possible fishing activity was detected using a speed filter for speeds smaller than or equal to 2.5 knots. Fig 4 presents the speed distribution for purse seiners, for all positions reported at least 10 km from shore and during day. It is noticeable that the majority of probable fishing activity happens with speeds in the range of 0 up to 5 knots, as indicated by the black distribution, and a second distribution peak appears for probable non-fishing activity (around 15 knots) in red.

The 2.5 knots speed threshold was chosen based on observations from the AIS data and the work of Bez *et al* [14]. The filtering based approach does not require machine learning, but the computation of extra features as described in the data pre-processing section.

## 4 Results

### 4.1 Trawler

As presented in Section 2.4, the solution proposed for trawling vessels is based on the HMM algorithm. As HMMs assume that the data is time dependent the analysis must consider the order of the points. Monte Carlo simulations are the only repeatable testing method that does not change this order. The objective of the repeated test is twofold: 1) to assess the HMM performance, and 2) to identify how many data points are required to correctly predict potential fishing activity.

**Fig 5:**
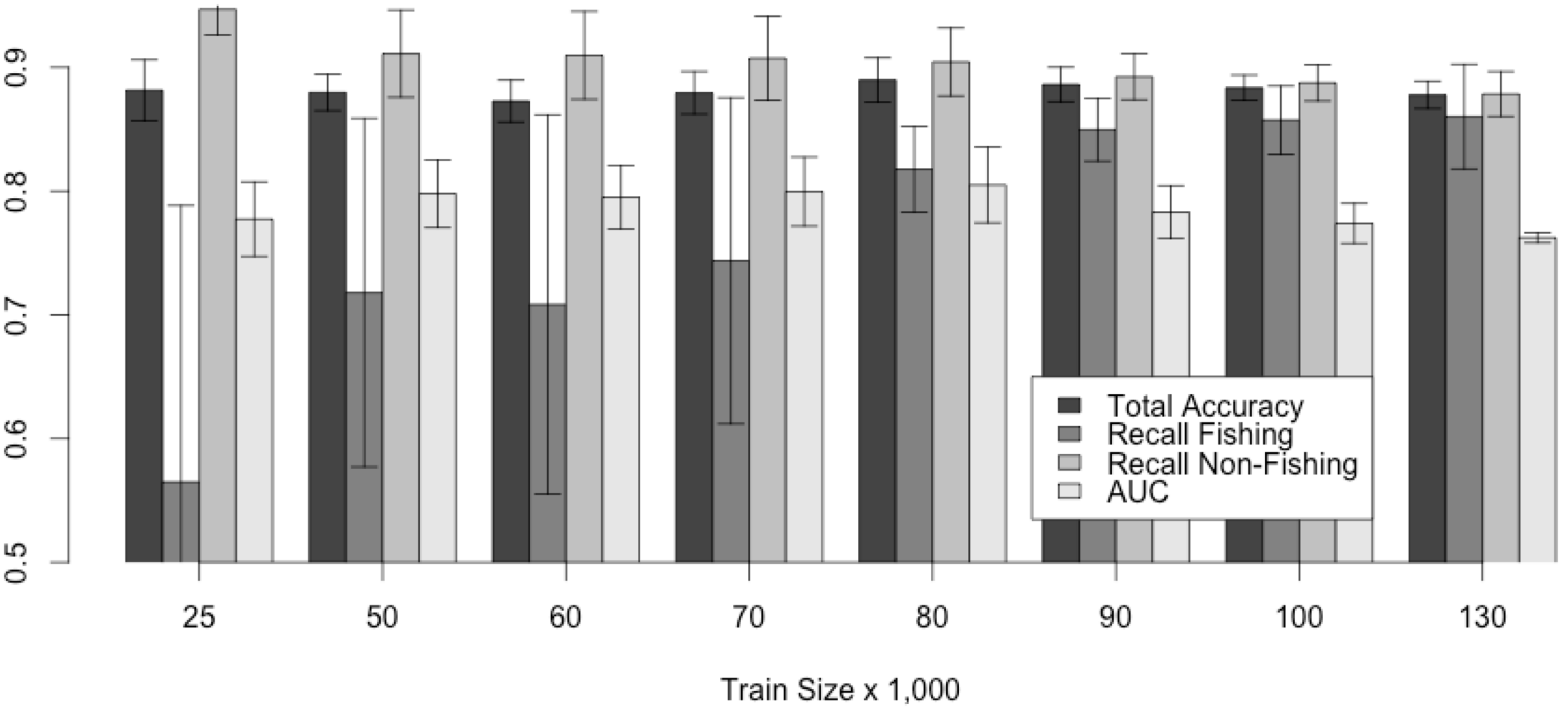
Accuracy/Recall measured for trawlers with HMM using Monte Carlo Simulation. Results do not consider the 10 km coastal distance threshold.

Fig 5 presents the Monte Carlo average results of the accuracy, recall and Area Under the Curve (AUC) for each class (fishing and not fishing). The HMM keeps the same average accuracy independently on the number of points available for training (close to 88%). The algorithm also improved the recall (defined as the percentage of relevant, correct fishing detections retrieved by the algorithm) of the fishing activity from 58% to above 85% when the algorithm was presented with more data for training, but also reduced the prediction of non-fishing activity from above an average 95% to 87% at the same time. This increase in the number of correct predictions of probable fishing activity indicates that the HMM may be overfitting, with higher number of points available for training. The overfitting aspect of the model can only be evaluated with tests on a different data set.

Despite the fact that the Monte Carlo simulations are a good indicator of the algorithm’s performance, they do not give the full information about how the algorithm will work with future data sets. To confirm the number of points necessary for training we tested the HMM against data from other parts of the world. The training was done on the 2013 North Pacific data, which contains 25,000 points. The test data was derived from the seven vessels operating across all oceans and years. The algorithm showed low accuracy results when trained with more than 25,000 data points, due to overfitting. This lies in the nature of the Monte Carlo test methodology: the more data available for training also means less data for testing, which reduces the variance (Fig 4). To avoid overfitting, it is advisable to train the HMM with fewer data points to increase its generalization. The results indicate that the HMM can be used to locate probable fishing activity for trawlers using tracks from different areas of the world with a median accuracy of 84%. There was little difference in accuracy for the two subtypes of trawling, pelagic/midwater (average accuracy 75%) and bottom (average accuracy 80.6%). The sensitivity to detect probable fishing activity (column Sensitivity in Table 1) shows a median of 93% and the respective specificity (the capability to identify probable non-fishing activity) a median of 68%.

**Fig 6:**
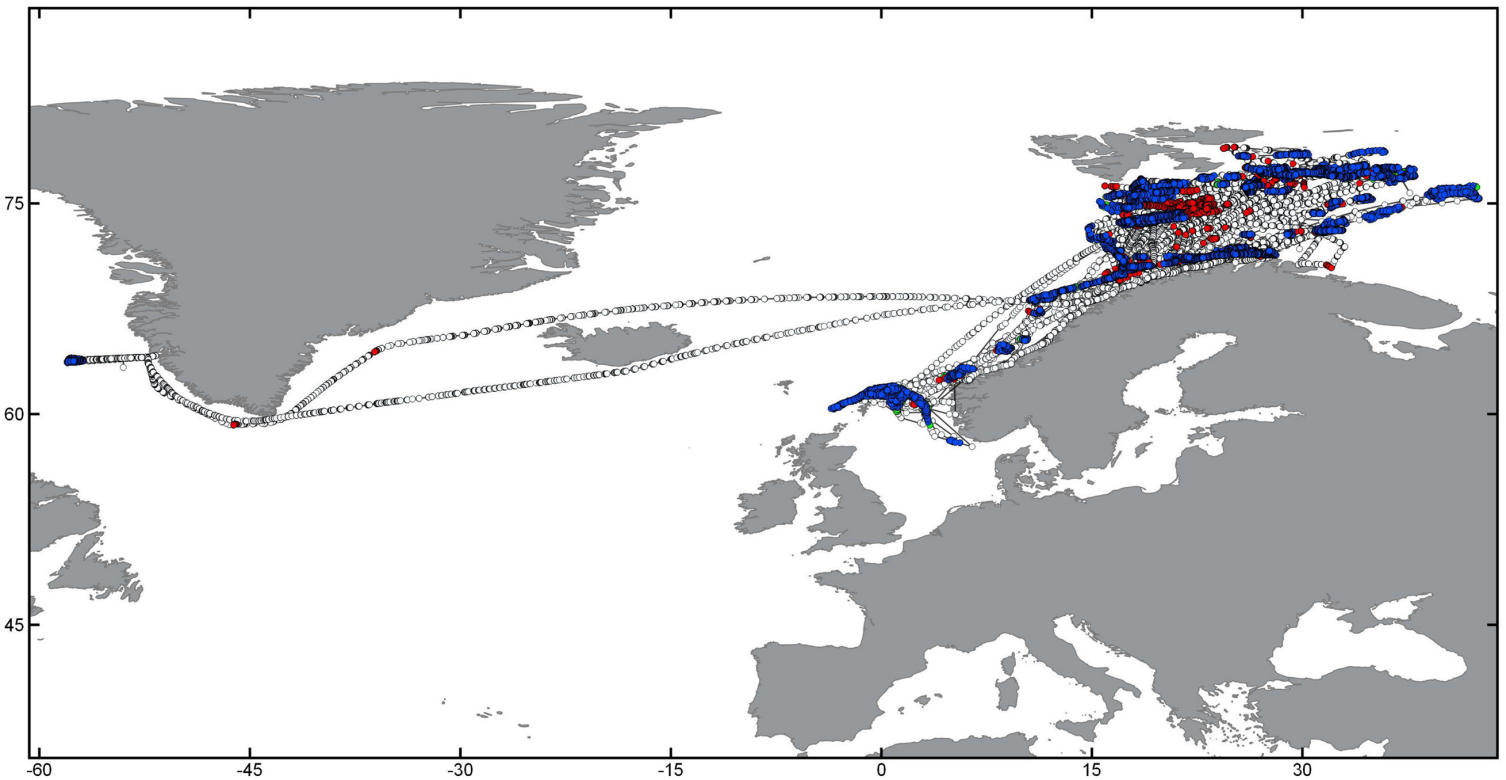
Comparison of the Hidden Markov Model algorithm results to the expert labels. Matching results for fishing activity presented in blue, expert labels in green and the algorithm’s fishing activity predictions in red. Empty circles represent non-fishing activity as identified by algorithm and expert. The track corresponds to vessel number 2 in Table 1. Map data by Natural Earth.

Fig 6 presents the results for track number two from Table 1, containing 254,323 points with a total accuracy of 84% and 69% specificity to detect probable fishing activity, as well 93% sensitivity of probable non-fishing activity detection.

As shown by [20], the sensitivity and specificity only concern the current model’s capability to classify the test instances, but they give no information about future algorithm performance. With support of the information provided in columns Prediction (F) and Prediction (NF), it is possible to confirm that the algorithm will have high probability to keep the same performance with unseen data.

### 4.2 Longliner

The 16 longliner vessels were tested independently as a mathematical model was fit to the data set and all the data was used for testing. The longliner database contains an average of 76% of movement patterns dedicated to assumed fishing activity. Table 2 summarizes the results of the longliner detection. In general, the median algorithm performance is 83%. As previously presented in Section 2.4, the columns Prediction (F) and Prediction (NF) shown in Table 2 indicate how well the algorithm will perform on unknown data sets. Results indicate that non-fishing activity (NF) will be correctly predicted in 87% of all cases with 57% accuracy and probable fishing activity (F) is identified correctly 77% of the time with an accuracy of 93%.

**Fig 7:**
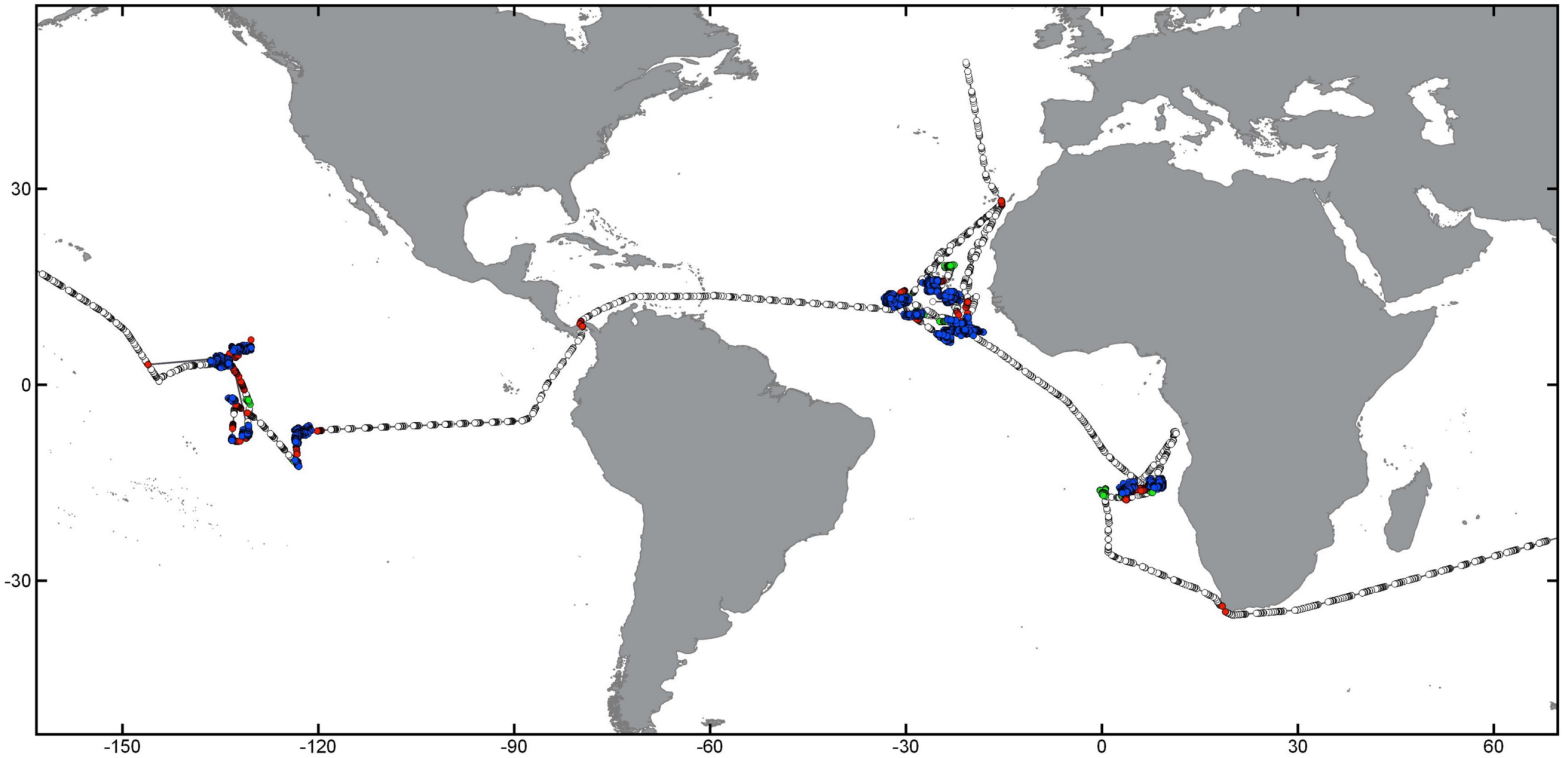
Results for longliner number 8 from Table 2(Accuracy: 89%). Matching results for fishing activity presented in blue, expert labels in green and the algorithm’s fishing activity predictions in red. Empty circles represent non-fishing activity as identified by algorithm and expert. Map data by Natural Earth.

**Fig 8:**
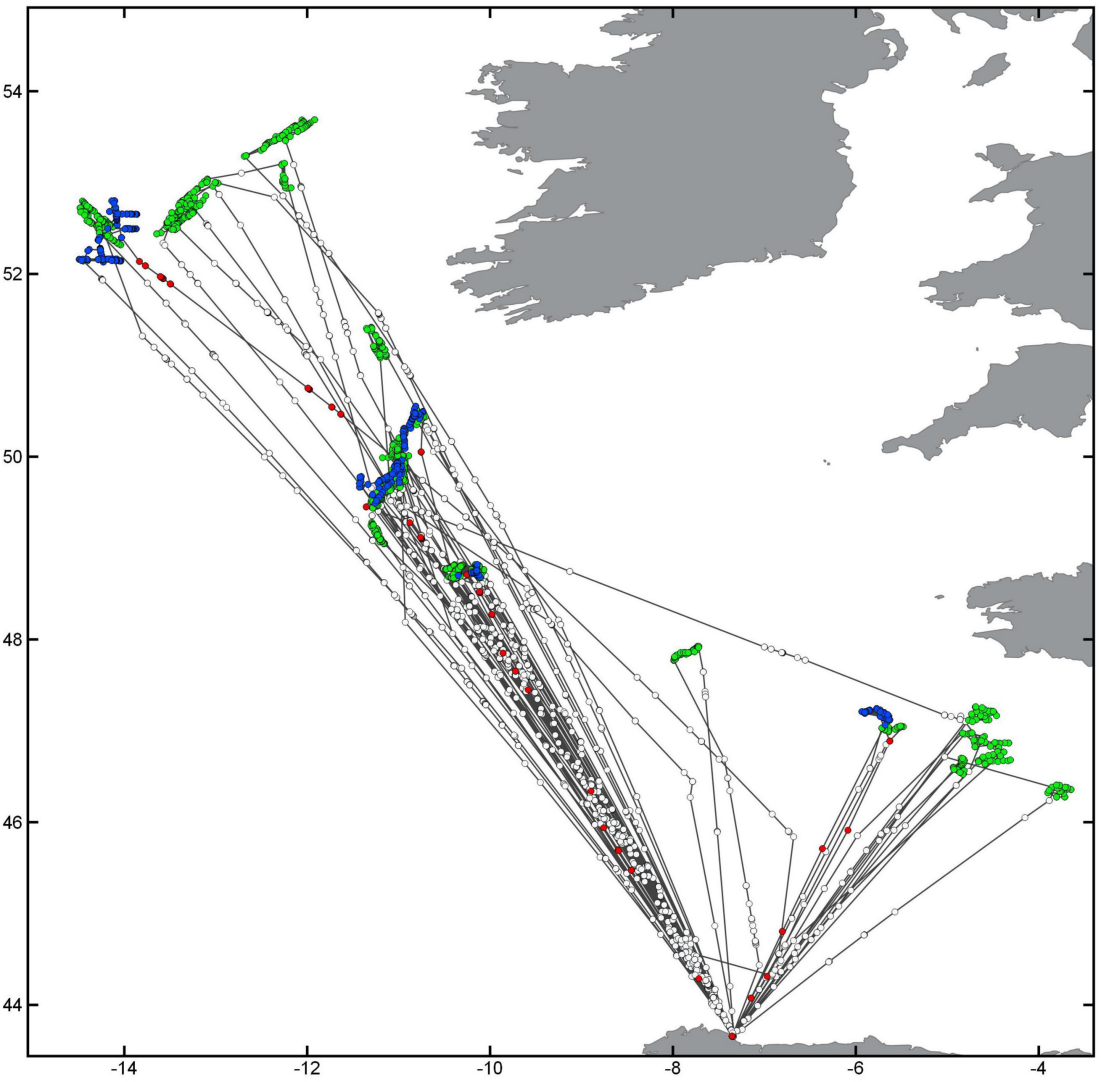
Results for longliner number 1 from Table 2 (Accuracy: 46%). Matching results for fishing activity presented in blue, expert labels in green and the algorithm’s fishing activity predictions in red. Empty circles represent non-fishing activity as identified by algorithm and expert. Map data by Natural Earth.

Figs 7 and 8 illustrate the results of the fishing prediction for tracks 8 and 1 in Table 2, respectively. These tracks were chosen because these vessels presented the highest and lowest accuracy results in the longliner data.

The last column presented in Tables 1 and 2 shows the results of a t-test between the algorithm’s predicted Fishing Effort (FE) and the expert’s labels. The FE is a measure to estimate how much time the vessel invests in fishing activity and is calculated in two steps: 1) periods of fishing activity for each vessel are tagged at the moments when a change from non-fishing activity to fishing and vice-versa occurred, and 2) for each individual period of fishing the time difference between the first and last AIS messages is calculated. A statistical difference between expert and prediction labels (*p* < 0.05) occurred only in one case (*p* = 0.05, Tables 1 and 2). This indicates that the algorithm is capturing the expert labeling for nearly all of the vessels evaluated. Two of the vessels were not part of the analyses as they did not have any fishing activity labeled by the expert.

### 4.3 Purse Seiner

Table 3 illustrates the results for the Purse Seiner filtering approach. The total median accuracy of the model is 97%, with a standard deviation of 1%. The filter median prediction capability of probable fishing and non-fishing activities is 97% and 94%, respectively. The main difference is in the sensitivity measure associated with probable non-fishing activity, with a median of 99% and a standard deviation of 1%. The specificity associated with probable fishing activity detection is around 71% with a standard deviation of 17%.

**Table 3:** Result for the purse seiner filtering approach. The seven vessels were randomly chosen from multiple parts of the world. NF stands for probable non-fishing and F for probable fishing events. Sensitivity is related with non-fishing detection, and specificity with fishing detection.

Track ID	Track Size	Accuracy	Prediction (NF)	Prediction (F)	Sensitivity	Specificity	AUC	% of Fish Activity
1	43457	0.95	1.00	0.02	0.95	0.82	0.89	0.05
2	170972	0.98	0.98	0.94	1.00	0.71	0.85	0.05
3	43369	0.96	0.96	0.95	1.00	0.36	0.68	0.02
4	18122	0.94	0.97	0.76	0.96	0.79	0.88	0.13
5	38596	0.99	1.00	0.91	1.00	0.89	0.94	0.03
6	51545	0.97	0.97	0.97	1.00	0.65	0.83	0.05
7	33484	0.97	0.97	0.96	1.00	0.64	0.82	0.05
**Median±SD**		0.97±0.01	0.97±0.01	0.94±0.34	0.99±0.01	0.71±0.17		

All these results indicate that the model is highly accurate to detect probable fishing activity when comparing to expert labels. The FE metric was not estimated for purse seiners as due to incomplete satellite coverage the total purse seining activity from starting to set the net to the finishing of the haul is rarely seen completely. Subsequently the filtering was designed to capture the hauling portion of the fishing activity only. This makes the FE metric unreliable to estimate the correct probable fishing time intervals. Instead, we present the AUC which is a common metric used in ML to inform on algorithm performance. The median AUC for the purse seiner is 0.85, with a standard deviation of 0.08, which also indicates the model is reliable to identify the minority class.

**Fig 9:**
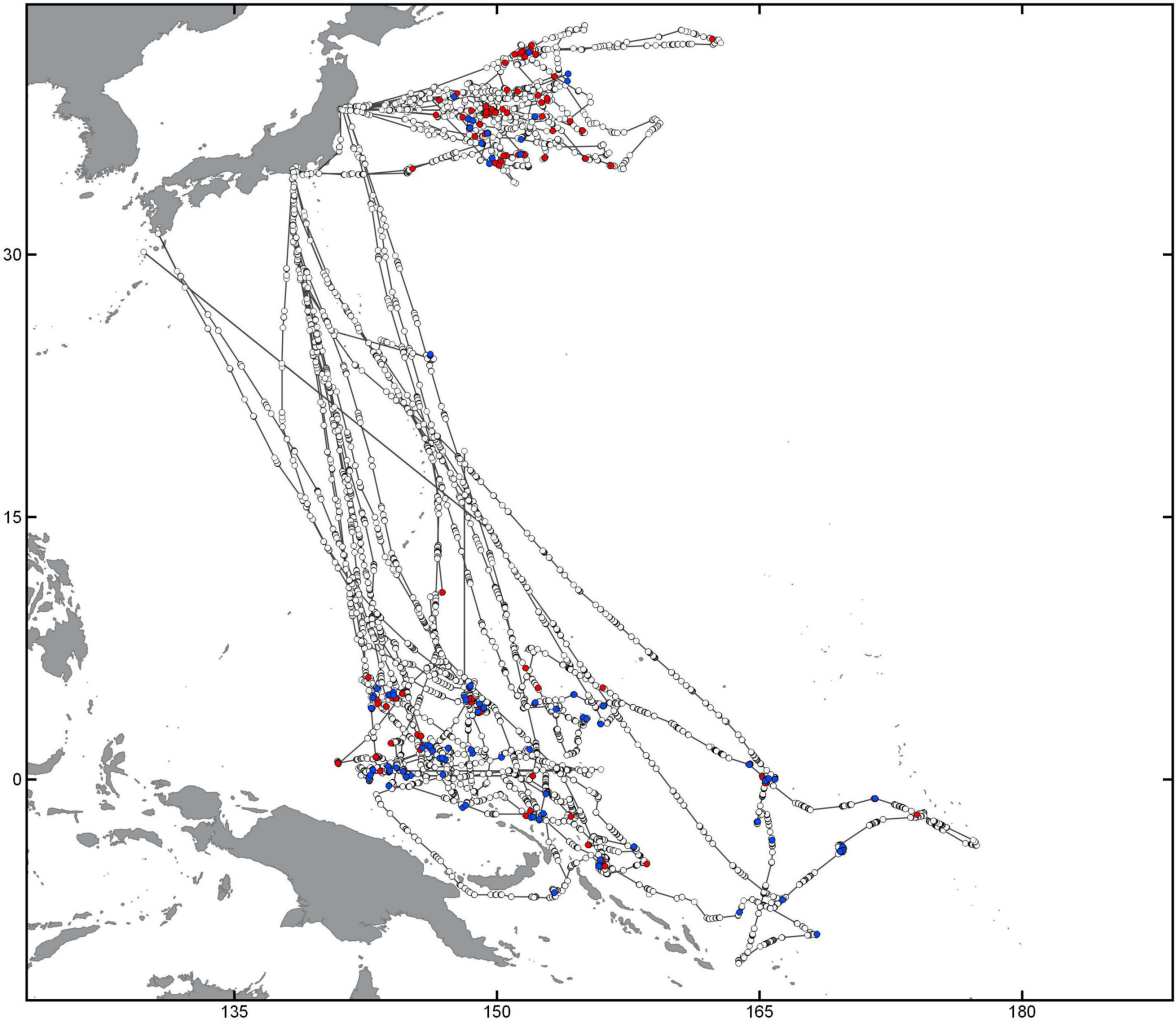
Comparison of the purse seiner algorithm results to the expert labels. Matching results for fishing activity presented in blue, expert labels in green and the algorithm’s fishing activity predictions in red. Empty circles represent non-fishing activity as identified by algorithm and expert. The track corresponds to vessel number 6 in Table 3. Map data by Natural Earth.

Fig 9 presents an example of the results found with the filtering technique proposed and corresponds to track number six in Table 3. The track contains 51,545 points and the filtering algorithm reached 97% total accuracy with nearly all probable fishing activity detected. The detection of false alarms is based on the expert’s labeling strategy, which classified speeds higher than 2.5 knots as fishing.

## 5 Discussion

The aim of this work was to develop automated methods to detect potential fishing behavior from different gear types based on S-AIS track data. As fishing activity for each gear type has its unique pattern and characteristics (compare Fig 1), we developed separate approaches tailored to the specific challenges associated with systematic differences in vessel behavior, speed distribution, and fishing time.

Results indicate that our machine learning and data mining approaches were able to correctly identify a very large fraction (83–97%) of probable fishing events compared to the expert labeled data. Importantly, our algorithms performed similarly well for different fleets in different regions operating from regional to global scales, making this a versatile tool to study the behavior of fishing fleets worldwide. While a number of previous studies have analyzed VMS data for vessel identification and tracking [6, 31, 32], S-AIS data have only been available for a few years and we are aware of only two published attempts to use it for detection and classification of vessel activities [4, 33]. Most previous work was done in engineering and computer science, largely either focusing on the technical aspects of system performance analyses mostly in the context of Maritime Situational Awareness [34–40] or on the computational challenge of complex analyses on big data and the combinations with other tools [33, 41]. While these studies form a valuable basis for the work presented here, no previous study has focused on a comprehensive solution to detect and classify fishing activities on a global scale using a distinction between gear types.

We presented three distinct machine learning, data mining and filtering approaches to detect potential fishing activity for trawlers, longliners and purse seiners, respectively. The method developed for trawlers is based on previous works [31] that showed a HMM is an effective way to predict fishing activity using vessel speed as the critical parameter. Our model works equally well for the two subtypes of trawling we accounted for, pelagic/midwater and bottom trawling. To establish the performance of the trawl algorithm, a Monte Carlo experiment was executed in which the algorithm performs increasingly better when the number of data points available for training increases. The objective to run Monte Carlo simulations is to assess the minimum number of points required to train a stable model, and confirm the model’s performance. Unfortunately, Monte Carlo simulations can suffer from overfitting, which is the case when tested models perform very well during the simulation but fail when new data is presented. Monte Carlo simulation results presented in Fig 5 indicate that models trained with 80,000 points would offer a more reliable and stable model, but when we tested these models against the data from various parts of the world, the accuracy reduced considerably. In order to confirm if the 80,000 points HMM was overfitting we tested HMM models with higher number of points for training. Results remained much below the accuracies reported by the Monte Carlo simulations, therefore confirming the overfitting assumption. As more generic models can be created with a reduced amount of data to avoid overfitting, we tested the HMM model with 25,000 points and results were very close to those reported by the Monte Carlo simulations for various parts of the world.

For longliner fishing activity detection no clear separation between fishing and non-fishing speeds exists. To detect probable longliner fishing activity, the development of an alternative algorithm was required. Since the proposed method is a Data Mining-based approach, all the data was used for testing, and the results indicate that the proposed method based on previous analyses of animal movement and habitat selection (see [15]) offers a good prediction level to detect a more complex type of fishing pattern. The main results are presented in Table 2 and indicate that the algorithm has good prediction capabilities with a median accuracy of 83%. One disadvantage of the method is the track segmentation algorithm, which requires defining the number of segments beforehand. Further work might consider methods to dynamically partition tracks.

Finally, the purse seiner approach uses another DM algorithm based on a filtering strategy. The filter designed is similar to the one used by McCauley *et al*. [42]. The algorithm filters the data assuming that purse seiners only fish during day, and that fishing activities are characterized by low speeds (lower than 2.5 knots). Due to this type of behavior, the filter results are well aligned with expert labels, indicating that the filtering approach is well suited to this type of vessel. A limitation of the filter is that it will not capture probable fishing activity with speeds above the 2.5 knots threshold. This assumption opens the possibility of future research to create techniques that are based on movement shape to detect possible fishing activity at higher speeds.

The labeling for all three methods is point-based, attaching a fishing or non-fishing label to the individual AIS vessel position records. On the basis of these labeled points, fishing time per area can be calculated on any given scale. Future work includes the partitioning of sequences of labeled points into sets for each gear type to adjust the output of the methods presented here to other units commonly used as measures of fishing effort. This can also include further information on the characteristics of fishing vessels as either transmitted with the AIS message or available through vessel registry data bases such as size, tonnage or engine power.

Overall, the algorithms are slightly more likely to detect potential fishing activity than expert labels, reflecting the conservative approach taken in labeling. As most groundtruthed data such as observer and logbook data is proprietary and often impossible to access, manual vessel activity labeling by an expert provides a workable solution. A possible next step to further improve the algorithms is to test and train them on data containing groundtruthed fishing activity recordings such as observer or logbook data if available at a resolution matching that for vessels carrying AIS transponders. This would eliminate any variability potentially introduced through the manual labeling and provide a more precise picture of vessel activities.

Despite their flexibility and versatility, the approaches presented here come with limitations and caveats. The biggest weakness is the structure of the S-AIS system itself: not all vessels carry S-AIS transponders and those who do can still tamper with or disable the transponders, or falsify positional or identification data. Manipulated data and switching-off events can be detected using specially designed algorithms, but more comprehensive legislation regarding the use of S-AIS may be needed to address these non-compliance issues on a broader scale, e.g. as suggested by McCauley *et al*. [42]. Another issue regarding S-AIS data is the limited satellite coverage, which at this point provides limited time and space windows for observation, and samples some regions better than others. This inherent problem, however, will be alleviated with the projected launching of several new AIS-enabled satellites in 2016.

The methods presented here were designed to stand on their own but they perform best on pre-processed AIS data, where wrong detections, noise and faulty out-of-bounds data (e.g. observations on land) are removed. There are efforts currently underway to develop better ‘de-spoofing’ algorithms that can reliably detect faulty or falsified AIS messages with inaccurate information. In combination with such pre-processing and de-spoofing algorithms, our methods will be applied within the framework of ‘Global Fishing Watch’ (http://www.globalfishingwatch.org), an open-access online-tool to detect and visualize fishing activity worldwide, with a launch planned for fall 2016. Furthermore, we caveat that algorithm performance varies with the quality of the track, giving better results for long tracks with high detection continuity. The three approaches presented here are designed to be applied for vessels with known gear type so the appropriate method can be chosen. While work on the identification of gear types used by fishing vessels is ongoing, our methods are only partly applicable for vessels using mixed gears and not for other gears than the three addressed here.

In conclusion, the approaches we have developed allow us to detect and identify potential fishing behavior for three main gear types with high accuracy and spatial resolution on a global scale. This opens up a new window of transparency, providing information on ocean uses not only to marine spatial planners and managers as well as the public, but also lays a foundation for future scientific research on vessel behavior for different gear types and sizes. One major challenge lies in expanding behavior identification to small-scale and artisanal fishing vessels which currently remain largely invisible in most tracking systems. Spatial-temporal analyses of long-term tracking data will offer valuable insights into fishing effort intensity and distribution in various areas such as nation’s exclusive economic zones, the high seas and areas of special interest like marine protected areas and other areas of biological or managerial interest.
