# Correction: Improving Fishing Pattern Detection from Satellite AIS Using Data Mining and Machine Learning

**DOI:** 10.1371/journal.pone.0163760

**Published:** 2016-09-22

**Authors:** Erico N. de Souza, Kristina Boerder, Stan Matwin, Boris Worm

There is an error in the caption of Table 2. Specifically, the descriptions of the results for Sensitivity and Specificity are switched (sensitivity linked to non-fishing detection instead of fishing detection and vice versa for specificity). Please see the corrected Table 2 and caption here.

**Table 2:** Performance measures for the 16 longliner vessels in different oceans.

Track ID	Track Size	Accuracy	Prediction (NF)	Prediction (F)	Sensitivity	Specificity	% of Fish Activity	Stat. Diff. Fish Effort
1	7935	0.46	0.95	0.25	0.35	0.93	0.70	0.29
2	25558	0.80	0.56	0.87	0.52	0.88	0.80	0.08
3	9642	0.65	0.85	0.57	0.45	0.91	0.71	0.62
4	35258	0.89	0.70	0.93	0.71	0.93	0.81	0.67
5	34993	0.87	0.82	0.89	0.66	0.95	0.79	0.28
6	42566	0.89	0.83	0.90	0.54	0.98	0.88	0.35
7	25287	0.76	0.81	0.73	0.59	0.89	0.68	0.67
8	96314	0.54	0.97	0.48	0.22	0.99	0.87	0.05 *
9	123686	0.89	0.89	0.90	0.81	0.94	0.67	0.54
10	128668	0.74	0.96	0.66	0.49	0.98	0.75	0.82
11	2070	0.54	0.81	0.50	0.20	0.94	0.86	0.80
12	1452	0.95	0.95		1.00	0.00	0.00	NA
13	12405	0.71	0.93	0.43	0.68	0.82	0.44	0.34
14	18169	0.86	0.80	0.87	0.55	0.96	0.83	0.67
15	6421	0.99	0.99		1.00	0.00	0.00	NA
16	2780	0.88	0.93	0.80	0.89	0.86	0.36	0.30
**Median**±**SD**		0.83±0.15	0.87±0.11	0.77±0.21	0.57±0.24	0.93±0.04		

NF stands for probable non-fishing and F for probable fishing events. Sensitivity is related with fishing detection, and specificity with non-fishing detection. The column Stat. Diff. Fish Effort shows the t-test statistical comparison (p-value) between the predicted fishing effort time calculated from the algorithm’s labels and the expert’s labels. Two of the vessels could not be measured because they did not have any labeled fishing activity. The asterisk indicates a significant difference.
